# Guidewire Retrieval Device as Bailout for Massive Coronary Thrombus in STEMI

**DOI:** 10.1016/j.jaccas.2026.108887

**Published:** 2026-07-15

**Authors:** Yan Wu, Xizhan He, Wenyuan Li, Zuyi Yuan, Yongbai Luo

**Affiliations:** a Department of Cardiology, The First Affiliated Hospital of Xi'an Jiaotong University, Xi'an, China; b Department of Cardiology, Shaanxi Provincial Sengong Hospital, Xi'an, China

**Keywords:** acute myocardial infarction, bailout technique, guidewire retrieval device, mechanical thrombectomy, thrombus burden, thrombus extraction

## Abstract

**Background:**

Patients with ST-segment elevation myocardial infarction (STEMI) and massive thrombus burden (TIMI flow grade 5) remain challenging to manage. Conventional aspiration thrombectomy often fails to achieve adequate thrombus reduction.

**Case Summary:**

A 52-year-old man presented with recurrent right coronary artery occlusion 5 days after initial intervention for acute inferior STEMI. After failed conventional aspiration, a guidewire retrieval device (Lawnest) integrated with an aspiration catheter was employed as a bailout. Optical coherence tomography confirmed significant thrombus reduction from TIMI flow grade 5 to grade 3, enabling successful drug-eluting stent implantation.

**Discussion:**

This case demonstrates a novel bailout technique using a coronary-specific guidewire retrieval device integrated with aspiration catheter via a hemostasis valve for refractory massive thrombus when conventional thrombectomy fails.

**Take-Home Message:**

Coronary-specific guidewire retrieval devices can serve as bailout techniques for refractory massive thrombus when integrated with aspiration catheters to minimize distal embolization risk.

## Visual Summary

**Figure:**
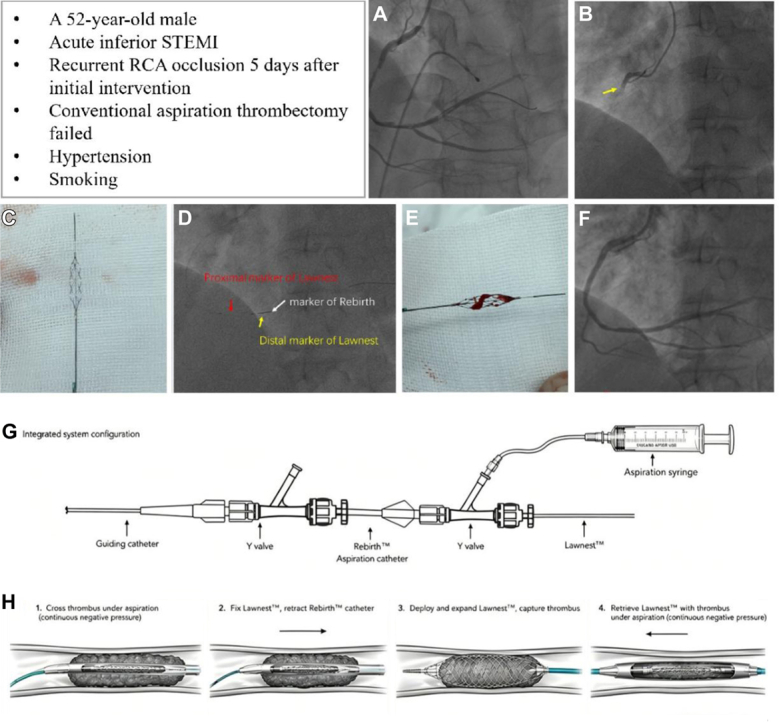

**Visual Summary:**
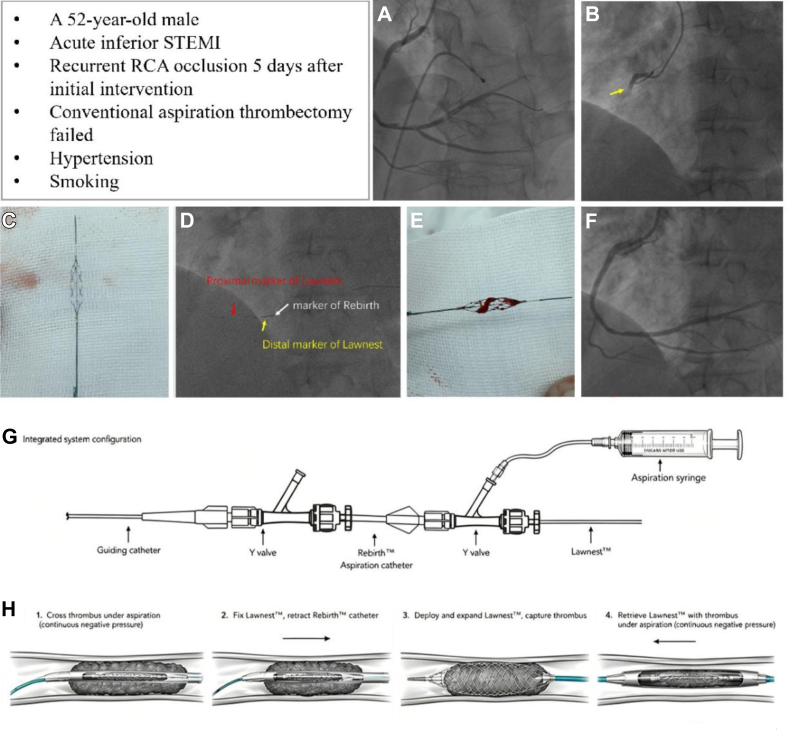
Guidewire Retrieval Device as Bailout for Massive Coronary Thrombus in STEMI (A) Angiography after first intervention showing multiple thrombi in RCA. (B) Repeat angiography 5 days later demonstrating RCA total occlusion with large thrombus burden (yellow arrow). (C) The Lawnest device in its fully expanded state. (D) Fluoroscopic image showing the system advanced to the mid-RCA. (E) Macroscopic appearance of thrombus captured by Lawnest device after retrieval. (F) Final angiography showing TIMI flow grade 3 without distal embolization. (G) Configuration of the integrated system. (H) Steps of coronary thrombectomy using the integrated system. RCA = right coronary artery; STEMI = ST-segment elevation myocardial infarction.

## History of Presentation

**Figure 1:**
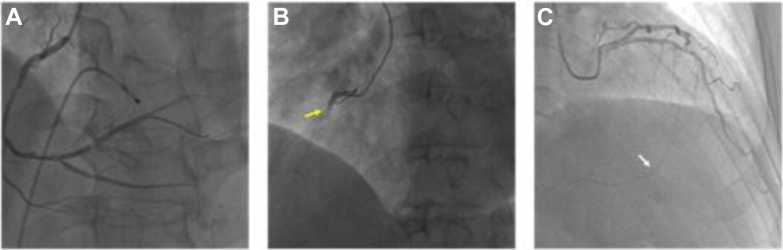
Initial and Repeat Angiographic Findings (A) Angiography after first intervention showing multiple thrombi in the RCA. (B) Repeat angiography 5 days later demonstrating RCA total occlusion with large thrombus burden (yellow arrow) and TIMI flow grade 0, consistent with TIMI thrombus grade 5. (C) Collateral circulation from the left circumflex artery (white arrow) providing Rentrop grade 2 flow to the distal RCA. RCA = right coronary artery.

A 52-year-old man with a history of active smoking presented to a local hospital with acute inferior ST-segment elevation myocardial infarction (STEMI). Emergency coronary angiography revealed total occlusion of the right coronary artery (RCA) with massive thrombus burden (TIMI flow grade 5), and angiography after first intervention showed multiple thrombi in the RCA (Figure 1A). Because of the massive thrombus burden, a stent was not implanted during the initial procedure given concerns for distal embolization and no reflow. The patient received intensive antithrombotic therapy including aspirin 300 mg loading dose followed by 100 mg daily, ticagrelor 180 mg loading dose followed by 90 mg twice daily, intravenous tirofiban infusion (25 μg/kg bolus followed by 0.15 μg/kg/min for 24 hours), and therapeutic anticoagulation with unfractionated heparin. He was then transferred to our tertiary care center for further management.

## Past Medical History

The patient had a history of hypertension that was well controlled.

## Investigations

Five days after the initial intervention, repeat coronary angiography was performed. The RCA demonstrated total occlusion with TIMI flow grade 0 flow and TIMI thrombus grade 5, indicating recurrent thrombotic occlusion despite intensive antithrombotic therapy (Figure 1B). Collateral circulation from the left circumflex artery provided Rentrop grade 2 collateral flow to the distal RCA (Figure 1C).

## Management

### Intervention and procedural details

A 7-F sheath was placed in the right femoral artery, and a 7-F JR4 guide catheter engaged the RCA. A BMW guidewire (Abbott Vascular) crossed the occlusion into the distal RCA. Multiple passes with a 6-F Rebirth aspiration catheter (Goodman) failed to adequately reduce the thrombus, with persistent angiographic TIMI thrombus grade 4 to 5.

**Figure 2:**
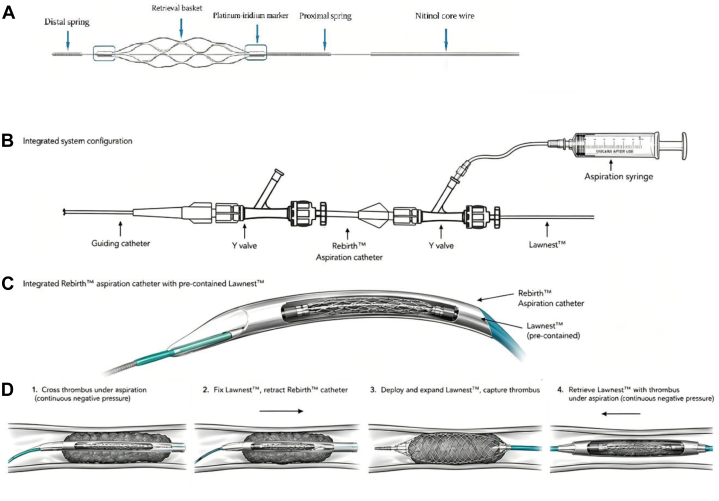
Integrated System and Retrieval Maneuver (A) Schematic illustration of the fully expanded Lawnest device showing its key components: distal spring, retrieval basket, platinum-iridium marker, proximal spring, and nitinol core wire. (B) Configuration of the integrated system. The Lawnest device is connected to a 6-F Rebirth aspiration catheter via a hemostasis valve (Y-valve) attached to an aspiration syringe. This assembly is advanced over the guidewire through the guiding catheter, which is equipped with a separate hemostasis valve. (C) Image showing the Lawnest device positioned inside the Rebirth aspiration catheter. (D) Steps of coronary thrombectomy using the integrated system: (1) The assembly is advanced across the large thrombus under active aspiration. (2) While maintaining continuous negative pressure through the hemostasis valve, the aspiration catheter is retracted to deploy the Lawnest basket within the thrombus. (3) Continuous aspiration is maintained; the expanded Lawnest basket engages and captures the thrombus. (4) The Rebirth catheter is carefully readvanced to recapture the Lawnest device, and the entire system is withdrawn with continued aspiration to prevent distal embolization.

Given the failure of conventional aspiration thrombectomy and the absence of commercially available dedicated coronary stent-retriever devices, a bailout strategy using the Lawnest guidewire retrieval device (APT Medical) was employed. The Lawnest device is a self-expanding, stent-like retrieval system specifically designed for coronary use, featuring a low delivery profile compatible with a 6-F aspiration catheter lumen (Figure 2A). When deployed, the basket expands to a diameter of 2.0 to 4.0 mm (self-adapting) with an effective length of 20 mm. The distal end is an atraumatic closed tip, and the proximal end is connected to a nitinol core wire. The device was integrated with the Rebirth aspiration catheter through a hemostasis valve (Y-valve), allowing continuous aspiration during retrieval maneuvers (Figures 2B to 2D).

**Figure 3:**
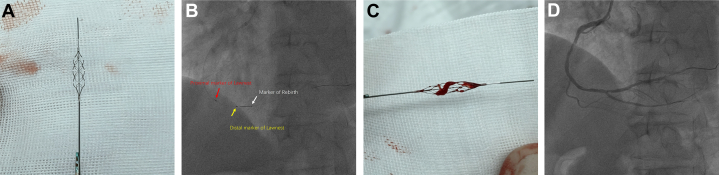
OCT Assessment and Final Results (A) Distal RCA showing acceptable lumen area with small residual thrombus (white arrow). (B) Proximal RCA demonstrating thin-cap fibroatheroma (asterisk) with moderate residual thrombus (red arrow) and reduced lumen area. (C) Final angiography showing TIMI flow grade 3 without distal embolization. (D) Final OCT demonstrating optimal stent expansion with minimal thrombus prolapse and good apposition. OCT = optical coherence tomography; RCA = right coronary artery.

The integrated system was advanced over the guidewire to the midsegment of the RCA with continuous aspiration maintained throughout (Figure 3B). The Rebirth catheter was then retracted to deploy the Lawnest device. After allowing 2 to 3 minutes for full expansion and thrombus engagement, the Rebirth catheter was carefully readvanced to capture the Lawnest device. The entire system was then withdrawn with continuous aspiration to prevent thrombus embolization. Visual inspection confirmed successful thrombus capture (Figure 3C). This maneuver was repeated 3 times, with each pass extracting visible thrombus fragments. Repeat angiography demonstrated significant reduction in thrombus burden, with TIMI flow grade 2 and downgrading from TIMI thrombus grade 5 to grade 3 (Figure 3D).

### Optical coherence tomography assessment

**Figure 4:**
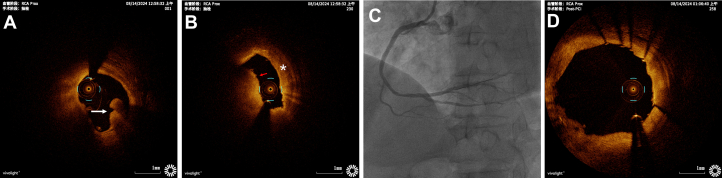
Coronary Thrombectomy With Lawnest Catcher (A) The Lawnest device in its fully expanded state. (B) Fluoroscopic image showing the system advanced to the mid-RCA. (C) Macroscopic appearance of thrombus captured by Lawnest device after retrieval. (D) Angiography after multiple retrieval passes showing significant thrombus reduction and restoration of TIMI flow grade 2 flow (downgraded from TIMI thrombus grade 5 to grade 3). RCA = right coronary artery.

Optical coherence tomography (OCT) was performed to assess vessel characteristics and residual thrombus burden. In the distal RCA segment, the lumen area was acceptable (approximately 8.5 mm^2^), with small amounts of residual thrombus adherent to the vessel wall (Figure 4A). The proximal RCA segment revealed thin-cap fibroatheroma with reduced lumen area (minimum lumen area: 4.2 mm^2^) and moderate residual thrombus burden (Figure 4B). These findings indicated that the retrieval device achieved substantial thrombus reduction (from TIMI flow grade 5 to grade 3).

### Stent implantation and final results

Based on OCT findings, a 4.0 × 23 mm drug-eluting stent (Xience, Abbott Vascular) was implanted in the proximal to mid RCA segment at 14 atm. Postdilation was performed with a 4.5 × 15 mm noncompliant balloon at 18 atm. Final angiography demonstrated TIMI flow grade 3 with no evidence of distal embolization or side branch compromise (Figure 4C). Repeat OCT confirmed optimal stent expansion (minimum stent area: 10.8 mm^2^) with good apposition, minimal thrombus prolapse through stent struts, and no significant edge dissection (Figure 4D).

## Outcome and Follow-Up

The patient tolerated the procedure well without complications. Postprocedural electrocardiogram showed resolution of ST-segment elevation. Transthoracic echocardiography performed 24 hours postprocedure revealed mild hypokinesis of the inferior wall with left ventricular ejection fraction of 52%. The patient was discharged on day 5 with dual antiplatelet therapy (aspirin 100 mg daily and ticagrelor 90 mg twice daily), high-intensity statin therapy, beta-blocker, and angiotensin converting enzyme inhibitor. At the 3-month follow-up, the patient remained asymptomatic, with no angina or heart failure symptoms. Repeat coronary angiography demonstrated patency of the RCA.

## Discussion

This case illustrates the persistent clinical challenge of managing STEMI patients with massive thrombus burden (TIMI thrombus grade 5). The initial decision to defer stent implantation given massive thrombus was reasonable, as immediate stenting in the presence of large thrombus carries significant risk of distal embolization and no reflow. However, the recurrent thrombotic occlusion despite intensive antithrombotic therapy highlights the complex pathophysiology of acute coronary thrombosis and the limitations of pharmacological therapy alone.

Whereas the TAPAS trial initially suggested benefit from manual aspiration thrombectomy in STEMI,^1^ the larger TOTAL trial definitively demonstrated no clinical benefit, and a potential increase in stroke risk.^2^ Consequently, routine thrombectomy is not recommended in current guidelines.^3^ However, these trials excluded patients with the highest thrombus burden, and the role of thrombectomy in selected cases with massive thrombus (TIMI flow grade 5) remains uncertain. In this case, conventional aspiration thrombectomy failed to achieve adequate thrombus reduction, necessitating an alternative approach.

The success of stent retrievers in acute ischemic stroke has prompted interest in their application for coronary thrombosis. Small case series have demonstrated the feasibility of stroke-derived devices in selected coronary interventions.^4^, ^5^, ^6^ Most recently, the RETRIEVE AMI trial demonstrated no device-related arterial complications or cerebrovascular events in the stent-retriever arm. Both stent-retriever thrombectomy and manual aspiration significantly reduced thrombus burden (stent-retriever thrombectomy: 12.8%, *P* = 0.016; manual aspiration: 13.0%, *P* = 0.003) compared with no modification (22.8%).^7^ Nevertheless, stroke-derived devices face challenges in coronary use, including size mismatch (deployed diameter 4-6 mm vs coronary dimensions 2.5-4.0 mm), incompatibility with simultaneous aspiration via standard 6-F catheters, and increased pushrod stiffness. Moreover, these devices remain investigational and are not yet approved or available for widespread clinical application.

The Lawnest coronary-guided retrieval device provides a valuable and feasible bailout strategy for refractory massive thrombus when conventional thrombectomy fails, with the following key advantages: 1) integration with standard aspiration catheters through a hemostasis valve, enabling continuous aspiration during retrieval; 2) compatibility with standard coronary guide catheters without the need for specialized equipment; 3) operation over a single guidewire, eliminating the requirement for parallel wire techniques; and 4) a design optimized for coronary vessel dimensions. In this case, the device successfully captured and extracted visible thrombus fragments, resulting in significant angiographic improvement. A recent case report described successful Lawnest thrombus retrieval using an extension catheter in an RCA with heavy thrombus, achieving TIMI flow grade 3 after conventional aspiration had failed.^8^ In our case, we extended this approach by integrating the Lawnest device with continuous negative-pressure aspiration, which successfully extracted visible thrombus fragments and resulted in significant angiographic improvement.

An important finding in this case was the OCT demonstration of residual thrombus after the use of the retrieval device. This observation highlights a crucial limitation: Even with advanced mechanical thrombectomy techniques, complete thrombus removal may not be achievable. This finding emphasizes that mechanical thrombectomy should be regarded as a debulking strategy rather than a method for complete thrombus elimination. The goal is to reduce the thrombus burden sufficiently to 1) restore antegrade flow, 2) reduce the risk of distal embolization during stenting, and 3) improve final angiographic and clinical outcomes. The decision to proceed with stent implantation despite residual thrombus was pragmatic, as prolonged attempts at complete thrombus removal carry inherent risks, including: 1) vessel injury from repeated device passes; 2) prolonged ischemia time; 3) increased contrast volume and subsequent nephropathy risk; and 4) potential for thrombus fragmentation and distal embolization. The final outcome of TIMI flow grade 3 without distal embolization represents a successful result despite incomplete thrombus removal, validating the efficacy of the debulking approach.

It is important to note that this report describes a technical approach in a single case and does not constitute a clinical series. The overall safety and efficacy of the Lawnest device require validation through prospective studies. This case report presents a single-center experience with a specific device, and results may vary depending on operator expertise or device type. The challenge of managing complex thrombotic conditions in acute myocardial infarction is not confined to any particular geographic region but constitutes a global clinical problem. Although this case was managed in China using a domestically developed device, the underlying principles and associated challenges are universally applicable. International collaboration and data sharing will be indispensable for advancing the field of mechanical thrombectomy for coronary applications.

## Funding Support and Author Disclosures

The authors have reported that they have no relationships relevant to the contents of this paper to disclose.

**Take-Home Message**

• Coronary-specific guidewire retrieval devices can serve as bailout techniques for refractory massive thrombus when integrated with aspiration catheters to minimize distal embolization risk.

**Equipment List:** 

Imaging • Optical coherence tomography (OCT) system (Shenzhen Zhongke Micro-Light Medical Equipment Technology)
Access • 6-F Glidesheath Slender hydrophilic coated introducer sheath (Terumo) • 5-F Optitorque TIG angiographic catheter (Terumo) • 7-F INT, SlideCath sheath introducer (Shanghai Kindly Medical Instruments) • 7-F JR4.0 guide catheter (Medtronic)
Percutaneous coronary intervention • BMW guidewire (Abbott Vascular) • 6-F Rebirth aspiration catheter (Goodman) • 6-F Lawnest guidewire retrieval device (APT Medical) • XIENCE Alpine everolimus-eluting coronary stent system 4.0 × 23 mm (Abbott Vascular) • NC Quantum Maverick balloon catheter 4.5 × 15 mm (Boston Scientific)
